# Toxoplasmosis and Polygenic Disease Susceptibility Genes: Extensive *Toxoplasma gondii* Host/Pathogen Interactome Enrichment in Nine Psychiatric or Neurological Disorders

**DOI:** 10.1155/2013/965046

**Published:** 2013-03-04

**Authors:** C. J. Carter

**Affiliations:** Polygenic Pathways, Flat 2, 40 Baldslow Road, Hastings, East Sussex TN34 2EY, UK

## Abstract

*Toxoplasma gondii* is not only implicated in schizophrenia and related disorders, but also in Alzheimer's or Parkinson's disease, cancer, cardiac myopathies, and autoimmune disorders. During its life cycle, the pathogen interacts with *~*3000 host genes or proteins. Susceptibility genes for multiple sclerosis, Alzheimer's disease, schizophrenia, bipolar disorder, depression, childhood obesity, Parkinson's disease, attention deficit hyperactivity disorder (*P*  from  8.01*E* − 05  (ADHD)  to  1.22*E* − 71) (multiple sclerosis), and autism (*P* = 0.013), but not anorexia or chronic fatigue are highly enriched in the human arm of this interactome and 18 (ADHD) to 33% (MS) of the susceptibility genes relate to it. The signalling pathways involved in the susceptibility gene/interactome overlaps are relatively specific and relevant to each disease suggesting a means whereby susceptibility genes could orient the attentions of a single pathogen towards disruption of the specific pathways that together contribute (positively or negatively) to the endophenotypes of different diseases. Conditional protein knockdown, orchestrated by *T. gondii* proteins or antibodies binding to those of the host (pathogen derived autoimmunity) and metabolite exchange, may contribute to this disruption. Susceptibility genes may thus be related to the causes and influencers of disease, rather than (and as well as) to the disease itself.

## 1. Introduction

The protozoan parasite *Toxoplasma gondii* (*T. gondii*) which causes toxoplasmosis, is primarily hosted not only in cats but also in mice, rabbits, dogs, farmyard and wild animals, and domestic fowl, and is transmissible to man [1–5]. It has been implicated in the pathogenesis of many diseases, most notably schizophrenia [6–8], but also with bipolar disorder [9] depression and suicide attempts [10]. There is also evidence from serological antibody studies that the parasite may be implicated in the aetiology of Alzheimer's and Parkinson's disease [11–13] and in certain epilepsies of unknown origin [14]. The parasite has also been implicated in a number of autoimmune disorders including antiphospholipid syndrome, cryoglobulinemia, ANCA-associated vasculitides, autoimmune thyroid diseases, systemic sclerosis, rheumatoid arthritis, inflammatory bowel disease, and systemic lupus erythematosus, possibly related to host/pathogen antigen homology [15, 16]. 

It has already been noted that several schizophrenia susceptibility genes are related to the *T. gondii *life cycle, as well as to that of other pathogens implicated in this condition (cytomegalovirus, influenza, rubella, and herpes viruses) [17, 18] and that in both Alzheimer's disease (herpes simplex, *Chlamydia pneumoniae*, *Helicobacter pylori*, and *Cryptococcus neoformans*) [19, 20] and multiple sclerosis (Epstein-Barr virus) [21], susceptibility genes are also related to the life cycles of suspect pathogens. In animal models, and without the aid of any gene variant, such agents can, per se, induce pathological features relevant to the disease process, for example amyloid deposition and tau phosphorylation (induced by herpes simplex, *C. Pneumoniae*, treponemas, *Borrelia burgdorferi,* and other spirochetes) [22–24], demyelination induced by various viruses [25], or dopaminergic overactivity in the case of *T. gondii *[26]. The H1N1strain of the influenza virus is also able to destroy neurones in the substantia nigra, provoking Parkinsonian symptoms in laboratory models [27]. Pathogens can thus be regarded as potential causes, likely acting in a gene dependent manner. Many such agents show a seroprevalence far above the incidence of the disease with which they are implicated; for example, *T. gondii *may infect 30% of the world's population [28] in comparison to a schizophrenia prevalence of ~1% [29], and, as is the case with genetic risk factors, conflicting epidemiological data have often cast doubt upon whether such pathogens can truly cause disease [30]. However, this situation also applies to *Helicobacter pylori*, which indubitably causes stomach ulcers and likely gastric cancer [31, 32], although not all of the many infected with this agent (~50% of the world population [33]) succumb to these conditions. Any causative effects of such agents in man must therefore be conditioned by other factors, among which are immunity and resistance to the pathogen; pathogen strain or the timing and severity of infection; other confounding environmental and medical factors as well as the susceptibility genes for each disease. The effects of risk promoting gene variants, which are also present in control populations, albeit in lower proportion, must also be conditioned by environmental and epigenetic factors, as well as by gene/gene interactions.

During its life cycle any pathogen interacts with hundreds of human proteins whose function can only be compromised by their diversion to the attentions of the invader. In addition, bacteria and parasites scavenge important metabolites from host cells or fluids and donate other compounds to the host which must react accordingly. Activation of the immune system and inflammatory defence, involving chemokines, cytokines and numerous other mediators are an evident consequence of any infection, as are the resulting fevers [34]. It has also been noted in many bioinformatics studies that pathogen proteins closely resemble our own, and that immune attack directed towards the pathogen may thus result in antibody cross-reactivity with human proteins. The development of pathogen-derived autoantibodies may also play a key role in this pathological scenario [18, 19, 21, 35–39]. 

As shown below, the hundreds of human proteins implicated in the *T. gondii *life cycle are highly enriched in the products of susceptibility genes for the numerous conditions with which this parasite has been associated, as well as for others where a link is not yet suspected. The human pathways deranged by the parasite are also relevant to each condition. Subsets of the extensive *T. gondii *host/pathogen interactome appear to be relatively specific for distinct diseases suggesting that they relate to the cause of the disease, and that they may be able to direct the attentions of the pathogen towards particular pathways, pathologies, and disease. 

## 2. Methods

Briefly, lists of several hundred susceptibility genes involved in eleven different diseases were compared with a list of several thousand host genes implicated in the *T. gondii *host/pathogen interactome. Any significant enrichment of interactome genes within susceptibility gene datasets (and *vice versa*) was identified by statistical analysis.

The genes and environmental factors implicated in the various diseases (Alzheimer's disease, attention deficit hyperactivity disorder, autism, bipolar disorder, chronic fatigue syndrome, depression, schizophrenia, multiple sclerosis, Parkinson's disease, anorexia, and childhood obesity) are listed at PolygenicPathways (http://www.polygenicpathways.co.uk/) and at sites therein (including the autism database at Mindspec (AutDB) [40], the Bipolar database at the University of Chicago [41], AlzGene, MSGene, PDGene and SZGene [42–45]). Genome-wide association data can be accessed at the National Human Genome Research Institute http://www.genome.gov/gwastudies/ [46].

Host/pathogen interactions for *T. gondii *and microarray data (mRNA expression changes in response to *T. gondii *infection) were collected by literature survey and are listed at http://www.polygenicpathways.co.uk/tgondii.htm. Pathway analysis of the human arm of this interactome was performed using KEGG mapper [47] http://www.genome.jp/kegg/tool/map_pathway2.html, and the results are posted at http://www.polygenicpathways.co.uk/keggtgondii.htm. These and various other files relating to the analysis are posted at http://www.polygenicpathways.co.uk/toxoplasmosis.htm.

## 3. Statistics

The human genome currently contains 26,846 genes, 2792 of which are contained in the *T. gondii *host/pathogen interactome. In any other dataset, one would expect 2792/26846 genes to be involved with the pathogen (10.4%). Similarly, for N susceptibility genes in any disorder, one would expect N/26846 to appear in the host/pathogen interactome, providing the expected numbers in each colliding dataset. The significance of differences between the observed and expected values was assessed using the chi-squared test. Statistical analysis for the enrichment of particular KEGG pathways within datasets was performed using the tools at the Consensus Path Database (CPDB) [48] developed by the Max Planck Institute for molecular genetics http://cpdb.molgen.mpg.de/CPDB. Overlapping gene sets were identified using the Venny tool [49] at http://bioinfogp.cnb.csic.es/tools/venny/index.

## 4. Results

### 4.1. KEGG Pathway Analysis of the *T. gondii*/Host Interactome

2792 proteins or mRNAs are involved in the host/pathogen interactome, approximating to 10% of the human genome. A summary of the KEGG pathway analysis of the human arm of this interactome is provided in Tables 1 and 2.

As might be expected, a high proportion of genes are involved in the immune system and in pathogen defence pathways. Many are also involved in the life cycle pathways of a number of viruses, bacteria, and other parasites (Table 1). These stem in part from the common immune and defence mechanisms not only related to the pathogens (chemokine and cytokine activation, etc.), but also related to common signalling networks. The involvement of dedicated bacterial and viral defence pathways in the interactome (NOD, RIG1, and cytosolic DNA-sensing pathways) is likely to impact upon viral defence, although in which direction is impossible to determine. Interestingly, *T. gondii *produces an interferon-like substance with antiviral activity [50]. The host intestinal microbiome also influences *T. gondii *and is also able to act as an adjuvant in response to *T. gondii *infection by stimulating dendritic cells that provide the immunostimulation necessary to combat the parasite [51]. Such effects and the shared pathways between pathogens highlight an important potential cross talk between elements of the microbiome.

Diverse pathogens are implicated in all of the diseases in this study, and many of the pathways traced out by the disease susceptibility genes, *per se*, (posted on the PolygenicPathways website) also involve multiple viral and pathogen life cycle and immune-related pathways.

A number of cancer-related pathways are highly represented in the *T. gondii *interactome (Table 1). While a recent study has suggested its involvement in brain cancer, based on a correlation between cancer mortality and *T. gondii *seroprevalence [52], the parasite is able to arrest the growth of other cancerous cells via stimulation of the immune response and inhibition of angiogenesis. Antitumour effects have been observed in relation to spontaneous mammary tumours, leukaemia, lung cancer, and carcinogen-induced tumours following injections of Toxoplasma antigen or viable parasites in laboratory animals or cells [53].

Several autoimmune and atopic disease networks are involved in the parasite interactome. A high *T. gondii *antibody seroprevalence (as well as to the cytomegalovirus and the Epstein-Barr virus) has been observed in systemic lupus erythematosus, and it has been suggested that antibodies raised to the pathogen may contribute to the autoimmunity characteristic of this condition via pathogen/host protein mimicry [16, 54, 55]. Conversely, *T. gondii *infection has been shown to prevent the development of lupus-related nephritis in rabbits [56], a factor perhaps related to the immunosuppressant properties of parasitic infection. Toxoplasmosis has been reported to decrease leukocyte, natural killer cell, and monocyte counts in men, while increasing the same in women, with reduced B-cell counts in both [57]. No references were found for relationships between toxoplasmosis and Type 1 diabetes, a pathway also figuring in the interactome. Prior *T. gondii *infection has been associated with poor outcome in heart transplant patients (allograft rejection) [58]. Toxoplasmosis and other infectious agents have also been linked to cardiac myopathy [59–62], and diverse pathways of which were concentrated in the *T. gondii *interactome. In relation to asthma, the hygiene hypothesis, linking a reduced incidence of childhood infections (in general) to the worldwide increase in asthma and other allergic conditions, may be related to the concentration of *T. gondii *interactome genes within the asthma pathway, although a positive correlation of *T. gondii *infection and asthma has also been noted in Sweden [63–65]. The parasite clearly has multiple effects on diverse immune-related networks as noted above, and such effects are likely to be both beneficial and nefarious. For example parasite-related immunosuppression may well be useful (but perhaps not advisable) in autoimmune diseases such as multiple sclerosis but might also be expected to favour other infections. 

Many of the more specific signalling networks within the interactome (Table 2) can be related to the general processes described above. While the MAP kinase pathway is involved in a multitude of functions, the JAK/STAT pathway is involved in cytokine signalling, also bridging cytokine activation to cancer pathways [66]. The calcium signalling pathway is also activated by many processes and more specifically by voltage or receptor-gated ion channels (and is relevant to the “channelopathies” implicated in autism, depression, bipolar disorder and schizophrenia, and in neurological disorders [67, 68]) or by processes modulating intracellular stores, while the phosphatidylinositol signalling system is also involved in the actions of multiple messengers. TGF beta regulates proliferation, apoptosis, differentiation, and migration (definition from KEGG). Calcium channel blockers, calmodulin antagonism, or extracellular calcium depletion diminish cellular invasion by the parasite [69, 70]. The P53 and growth factor signalling networks (ErbB, VEGF) can be cancer related, while insulin signalling is evidently related to diabetes. PPAR receptors control the transcription of many genes especially those related to fatty acid metabolism, but also those involved in cell proliferation and differentiation [71]. These and other pathways control a host of processes from embryonic differentiation to cellular death and apoptosis, and many metabolic pathways that are too numerous to individually review. 

In relation to the diseases that are the object of this study, the Alzheimer's and Parkinson's disease pathways were both represented, as were the complement, PPAR, and terpenoid (cholesterol synthesis) pathways relevant to Alzheimer's disease [72], and the ubiquitin pathway relevant to Parkinson's disease and other degenerative disorders [73]. Erbb signalling is highly relevant to the control of peripheral and central myelination [74], and thus to multiple sclerosis and Alzheimer's disease, but also to a range of psychiatric disorders including autism, anorexia, ADHD, bipolar disorder, depression, and schizophrenia [75]. Myelin is exquisitely sensitive to oxidative stress and glutathione depletion (c.f. glutathione pathways), and the glutathione precursor N-acetylcysteine has been shown to be of benefit in a number of psychiatric disorders [76–80]. The diverse neurotransmitter pathways and many signalling networks are also relevant to most of these conditions. Rather than single out any particular pathway from this extensive dataset (Tables 1 and 2), suffice it to say that parasitic infection has massive effects upon a variety of host signalling networks, metabolic pathways, and processes. These are nevertheless relatively selective, in the sense that certain pathways are more affected than others. In addition, within each disease dataset, the spectrum of pathways within the overlapping datasets is distinct and biologically relevant, as detailed below. 

### 4.2. Enrichment of Interactome Genes within Susceptibility Gene Datasets (Table 3)

*T. gondii *interactome genes were significantly enriched in the susceptibly gene datasets for all diseases with the exception of anorexia and chronic fatigue and represented from ~13% (autism) to 33% (multiple sclerosis) of the total number of susceptibility genes analysed, with enrichment values from 1.08 to 2.83 fold the expected number (Table 3). For schizophrenia, the fold enrichment (interactome genes in susceptibility gene dataset) of 2.03 compares with a recent meta-analysis of *T. gondii *seroprevalence studies providing an odds ratio (OR) of 2.71 [81]. A further meta-analysis showed significant associations of schizophrenia with infections by human herpesvirus 2 (OR = 1.34), Borna Disease Virus (OR = 2.03), human endogenous retrovirus W (OR = 19.31), *Chlamydophila pneumoniae* (OR = 6.34), and *Chlamydophila psittaci* (OR = 29.05), including values far in excess of those for any gene [82]. For schizophrenia at least, these data and ample evidence from epidemiological and animal behaviour studies [83–85] firmly advocate toxoplasmosis as a significant cause of the disease, in those with a particular genetic constitution. The ability of the parasite to manipulate dopaminergic metabolism (via its own tyrosine hydroxylase) [86] and the involvement of NMDA receptor (e.g., glutamatergic signalling and long-term potentiation), serotonin, or cannabinoid-related signalling networks within the interactome is relevant to the drug-induced psychosis associated with the amphetamines, LSD, cannabis, or phencyclidine (see [87]). Dopamine also increases the number of *T. gondii *tachyzoites in cultured fibroblasts suggesting that neurotransmitters may also be able to manipulate the parasite [88]. 

For each disease, and across diseases, the types of susceptibility genes influenced were distinct and relatively selective for each disease. This was assessed in two ways: firstly by statistical analysis of the enrichment of KEGG pathways in each overlapping *T. gondii *interactome/disease dataset and secondly by a comparison of individual shared and specific overlapping interactome/disease genes across four diseases (the maximum possible using the Venny tool). The diseases analysed in this way were Alzheimer's disease and multiple sclerosis, bipolar disorder, and schizophrenia.

### 4.3. Overlapping Interactome/Susceptibility Genes Common and Specific to Four Diseases (Table 4, Figure 1)

**Figure 1:**
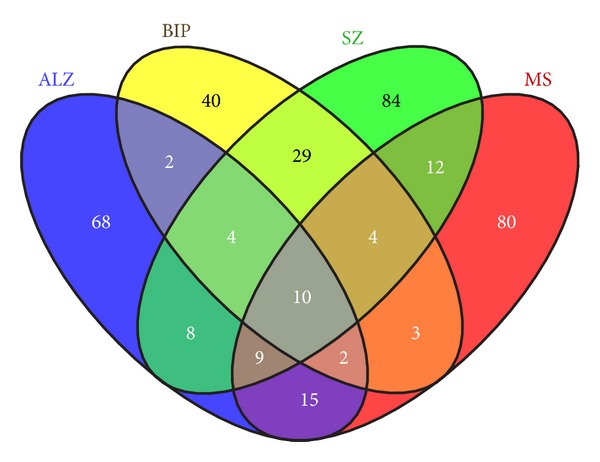
The Venn diagram illustrates the number of susceptibility genes (all within the *T*.* gondii*/host interactome) that are common or specific to various permutations of Alzheimer's disease (Alz), Bipolar disorder (Bip) Schizophrenia (SZ) ormultiple sclerosis (MS) (see Table 4 for genes in each section).

The permutations of genes common or specific to the various chosen diseases (Alzheimer's disease, bipolar disorder, schizophrenia, and multiple sclerosis) are shown by the Venn diagram Figure 1 summarised in Table 4. All of these genes are members of the host/pathogen interactome. Several immune/cytokine and oxidative stress related genes, with different identities, but similar roles, appear as common risk factors across various permutations of diseases, which are all characterised by immune activation [89–91] and oxidative stress [92, 93]. 

Bipolar disorder and schizophrenia share many common genes, risk factors, endophenotypes, and subpathologies, and interactome genes relevant to certain of these are related to circadian rhythm, dopaminergic and glutamatergic neurotransmission, growth factors, and signalling networks as highlighted in previous reviews [75, 94, 95].

After sifting through these common subsets, the overlapping *T. gondii *interactome/susceptibility genes specific to each disease are remarkably relevant to the key primary pathologies in each. They include APP processing, cholesterol and lipoprotein function, complement and immune related genes, and oxidative stress, apoptosis and ubiquitin genes in Alzheimer's disease [96–100]. In bipolar disorder, monoamine/GABA, signalling, adhesion, and ion transport genes are highlighted (see above and [101–103]) while in schizophrenia, monoamine/glutamate/neuregulin neuronal development and associated signalling related genes figure prominently, along with those related to adhesion, oxidative stress, and immune activation (see above). In multiple sclerosis, almost the entire common dataset is related to immune function and associated signalling pathways, that are relevant to the autoimmune aspects of the disease [104, 105], with a limited number of genes related to oxidative stress and apoptosis.

While the evidence for an involvement of toxoplasmosis in psychiatric disorders is relatively strong, there is less work either in the human condition or in animal models in the case of neurological disorders, such as Alzheimer's or Parkinson's diseases or multiple sclerosis. Toxoplasmosis has, however, been associated with a loss of grey matter density in schizophrenic patients, but not in controls, suggesting an influence on degenerative components [8]. *T. gondii *infection may not always be deleterious. For example, it inhibits the development of arthritis in mice deficient in the interleukin receptor antagonist (IL1RN) [106]. *T. gondii *infection is also able to reduce infarct size in focal cerebral ischaemia in mice, an effect attributed to the ability of infection to increase the expression of nerve growth factor, as well as that of anti-inflammatory cytokines and of glutathione and oxidative stress protective genes, while reducing the expression of proinflammatory cytokines [107]. 

Parasites have learnt to live with us for many millennia, and their immunosuppressant effects appear to be a relatively common defence mechanism. Indeed, the use of helminths (parasitic worms) has been suggested in a number of autoimmune settings including irritable bowel disease and multiple sclerosis [108]. A clinical trial with helminth egg infection (*Trichuris Suis* Ova) in autism is also listed at http://clinicaltrials.gov/, based on anecdotal reports of effectiveness in relation to certain symptoms. The preponderance of immune related host/pathogen genes in the multiple sclerosis dataset (and to a lesser extent within other datasets) may be related to these potentially beneficial effects, although the clinical use of *T. gondii *would be contraindicated by its malevolence directed elsewhere. 

### 4.4. KEGG Pathway Analysis of the Overlapping Datasets Specific to Each Disease (Tables 5 and 6)

#### 4.4.1. Immune and Pathogen Defence Pathways Common to Most Diseases

The KEGG pathways influenced by *T. gondii *(restricted to the overlapping interactome genes within each disease dataset) are posted at http://www.polygenicpathways.co.uk/toxoplasmosis.htm, and the CPDB enrichment analysis depicted in Tables 5 and 6. These tables report only the significantly enriched pathways, but many others figure within these overlapping datasets. In all diseases, except for ADHD and anorexia, the significantly enriched subsets involved immune or defence related pathways. For the most part (autism, childhood obesity, depression, bipolar disorder and schizophrenia, Alzheimer's and Parkinson's disease, and multiple sclerosis), the bacterial defence NOD signalling network was involved, while the similar Toll pathway was more restricted (Alzheimer's and Parkinson's disease and multiple sclerosis, bipolar disorder, and schizophrenia). The RIG1 and cytosolic DNA-sensing pathways recognise viral nucleic acids. The RIG-1 pathway was significantly enriched in multiple sclerosis and schizophrenia, while the cytosolic DNA-sensing pathway was enriched in Alzheimer's and Parkinson's disease as well as in multiple sclerosis and schizophrenia. Diverse pathogen life cycle pathways were enriched in all but the ADHD and anorexia datasets. 

#### 4.4.2. Childhood Obesity and Anorexia

There are few studies relating either obesity or anorexia to toxoplasmosis in man, although both anorexia or subsequent partial weight gain postinfection, as well as hypermetabolism have been associated with *T. gondii* infection in laboratory and farm animals [109–111]. 

The only significantly enriched pathways common to the *T. gondii* interactome in anorexia all relate to neuronal systems (dopamine, serotonin, and addiction pathways). 

In childhood obesity, a number of autoimmune related pathways were highlighted, as well as the Alzheimer's disease pathway, pathways related to PPAR signalling (regulating fatty acid metabolism), and glycerolipid metabolism. A recent review has highlighted the risk promoting effects of midlife obesity (and several other preventable risk factors) in relation to Alzheimer's disease [112]. The childhood obesity epidemic, fuelled largely by dietary and sedentary culture [113], has been associated with an increased risk of affective disorders in adulthood [114] and has also led to an increased incidence of a number of diseases in young children (dyslipidemia, carotid artery atherosclerosis, cardiac problems, hypertension, the metabolic syndrome, and diabetes and fatty liver disease) [115–118] that were previously the reserve of old age. Many of these are also risk factors for Alzheimer's disease and are able, *per se*, to increase cerebral beta-amyloid deposition in laboratory models, perhaps a herald for the unwelcome imminence of dementia in young adults. 

Diet, including saturated fat [119, 120], affects the microbiome, and a recent study has shown that, in infants fed formula or breast milk, changes in the gut microbiome can alter the expression of genes related to the innate immune system [121]. This microbiome/immune link may be important in the development of inflammation and metabolic diseases [120]. There do not appear to have been any microbiome studies in relation to *T. gondii*. However, the parasite scavenges host cholesterol, while host fatty acids and low-density lipoproteins stimulate a *T. gondii *acyl-CoA, cholesterol acyltransferase, which then provides cholesteryl esters that the parasite needs for its survival [122]. Fatty diets would certainly be expected to impact upon the success of this parasite, which in turn must influence the lipid metabolism of the host. Indeed, *T. gondii* infection may even possess beneficial effects in hypercholesterolaemic conditions in mice, reducing the development of atherosclerosis via cholesterol and lipoprotein scavenging effects [123]. 

Pathway correlates such as these of course predict relationships but not directionality, which can only be imputed by prior knowledge and future research. Certain of the pathways common to the *T. gondii* interactome and obesity (and to Alzheimer's disease, see below) could well reflect a beneficial component of parasitic infection. 

#### 4.4.3. Attention Deficit Hyperactivity Disorder and Autism

No clinical studies have specifically linked ADHD or autism to toxoplasmosis, although hyperactivity, modified social interactivity, and sensorimotor effects are features of infection in mice that are of relevance to both conditions [124–126].

In ADHD, the primary common emphasis was on the calcium signalling pathway to a number of metabolic pathways: phenylalanine and tyrosine (DDC and MAOA), tyrosine, histidine (DDC, HNMT, and MAOA) tryptophan (ACAT1, DDC, and MAOA), and unsaturated fatty acid synthesis (FADS1 and FADS2) and to neurotransmitter pathways (cocaine addiction and ligand/receptor interactions). This is a relatively small dataset, but it highlights an important distinction for bacteria or parasites, which, unlike viruses, participate in substrate and metabolite exchange with the host, enabling a much greater effect on metabolic pathways. This influence may be particularly relevant to the reported risks and benefits of various types of diets in many diseases, and in particular, saturated and unsaturated fats [127]. 

In autism, various cardiomyopathy pathways were enriched in the overlapping dataset. Autistic components have been observed in a number of cardiomyopathy disorders (MELAS and Timothy syndromes and Danon disease) [128–130]. *T. gondii *seropositivity has also been associated with cardiomyopathy [60]. Cellular adhesion and the extracellular matrix play a key role in brain development and in autism [131, 132], and these pathways were the only significantly enriched “processes” in the overlapping dataset. VEGF signalling, dopamine, serotonin, and addiction pathways, but no metabolic pathways, were also enriched. Serum VEGF levels have been reported to be reduced in severely affected autism cases [133]. 

#### 4.4.4. Depression and Bipolar Disorder

Although perhaps less evident than with schizophrenia, toxoplasmosis has nevertheless been associated with prenatal depression, depression, bipolar disorder, and with a history of suicide attempts in recurrent mood disorders [9, 10, 134, 135].

In depression, as well as immune, defence, and diverse pathogen related pathways, autoimmune diseases, hypertrophic cardiomyopathy, rheumatoid arthritis and osteoclast differentiation, cancer pathways, and Alzheimer's disease were overrepresented in the overlapping dataset. Depression and arthritis have been reported as comorbid conditions [136], and prior depression is a significant risk factor in both cardiac conditions and Alzheimer's disease [137, 138]. Numerous studies have implicated the VEGF pathway is relevant to depression and to the mechanism of action of antidepressants [139]. With regard to transforming growth factor, TBF-beta, an anti-inflammatory cytokine, an imbalance of pro- and anti-inflammatory cytokines has been observed in major depression studies [140]. Neuronal pathways primarily concerned reward/addiction, glutamate, dopamine, serotonin, and cannabinoid networks. An overrepresentation of phenylalanine and tryptophan metabolism is also relevant. The circadian clock pathway, which was also over-represented, plays a key role in depression and related disorders [141]. In drosophila, the circadian clock regulates the phagocytosis of bacteria [142], and within its many functions are the control of the immune system [143]. Unsaturated fatty acid metabolism again figured in this group, and the general benefits of modifying saturated/unsaturated fat ratios in diet are increasingly recognised, including in the area of psychiatry [144].

The overlapping dataset in bipolar disorder also concerned immune related pathways, several autoimmune disease networks (Type 1 diabetes, arthritis (and osteoclast differentiation), graft-versus-host disease, and allograft rejection), and a number of pathogen life cycle pathways. In relation to cancer pathways, slight increases in overall cancer risk have been reported in both bipolar disorder and schizophrenia, which appear to be gender dependent [145]. In relation to the Alzheimer's disease pathway (which independently figures in all KEGG pathways related to susceptibility genes alone in most of these disorders), prior psychiatric illness has been shown to be generally associated with an increased risk of developing dementia [146]. Common pathological features across many psychiatric disorders and Alzheimer's disease also include white matter changes related to demyelination [147, 148]. Many of the stressors involved in these conditions (starvation, viruses, infections and fever, cytokines, oxidative, and endoplasmic reticulum stress) converge on a pathway that ultimately inhibits translation initiation and protein synthesis. This network is counterbalanced by growth factors and neurotransmitter influences that affect plasticity and growth and is particularly important in regulating oligodendrocyte viability, myelination, and synaptic plasticity [149] (c.f. the neurotrophin pathway within this dataset and related glutamatergic and growth factor signalling networks in others). Neurotransmitter networks within the overlapping bipolar/interactome are predominantly related to dopamine and reward pathways and to tyrosine, phenylalanine, and tryptophan metabolism.

#### 4.4.5. Schizophrenia

The link between schizophrenia and toxoplasmosis is perhaps the strongest in relation to published studies [6, 82, 150–152], and of particular relevance is the parasite's ability to increase cerebral dopamine levels (see above). In this respect, the overlapping interactome/gene dataset was enriched in dopaminergic pathways, and also in those related to serotonergic and glutamatergic transmission as well as cocaine and amphetamine addiction. As in most cases autoimmune and atopic diseases, which are commonly associated with schizophrenia, were well represented. In many autoimmune conditions the link with schizophrenia was positive and gender specific, while an inverse association between schizophrenia and rheumatoid arthritis was observed [153, 154] (c.f. the concentration of osteoclast differentiation pathways in this dataset). Gluten sensitivity (characterised by antibodies to a gluten constituent protein, gliadin), other food antibodies, and celiac disease have also been associated with schizophrenia. Food antigens in schizophrenic patients have been shown to be correlated to the presence of *T. gondii *antibodies. Interestingly, the antiparasitic agent artemisinin reduces the titre of antibodies to gliadin in a subset of schizophrenic patients, and these observations testify to the ability of the parasite to modulate immune function (and perhaps the antigenicity of other proteins). However, artemisinin did not reduce the titre of antibodies to *T. gondii*, nor did artemisinin (as add on therapy) have significant effects on symptomatology [155–157]. Artemisinin and its analogues are known to produce neurotoxic effects in laboratory models, an effect possibly linked to excitotoxicity and oxidative stress [158, 159], and clearly more suitable agents are needed in the research domain. The overlapping dataset also included significant enrichment of adhesion molecule, glutathione, and growth factor and related signalling pathways (VEGF, MAPK, and Wnt, but not ERBB signalling, although this pathway is affected by *T. gondii*). The PPAR network was also enriched in this dataset and is relevant in relation to the inflammatory arm of this pathway and to the ability of the pathway to regulate cholinergic and dopaminergic function [160, 161]. With regard to the cancer pathways in this dataset, schizophrenia has been associated with a reduced cancer incidence, but with no familial explanation, suggesting a nongenetic reason that may conceivably be related to the abilities of *T. gondii*, and other relevant pathogens, to favour the promulgation of one disease, but perhaps protect against another. In relation to the overlapping Alzheimer's disease pathway within this dataset, the association of prior psychiatric illness with dementia has already been mentioned [146].

### 4.5. Neurodegenerative Disorders

#### 4.5.1. Parkinson's Disease

There are only limited human seroprevalence studies and no apparent animal studies specifically in relation to the substantia nigra, linking toxoplasmosis to Parkinson's disease [11, 12]. Nevertheless the overlap between the *T. gondii* interactome and susceptibility genes figures certain key pathways that may merit further research.

The interactome/genetic overlap for significantly enriched pathways in Parkinson's disease in relation to neurotransmission was restricted to dopaminergic systems, and a number of key genes including those of the mitochondrial respiratory chain (ATP6, CYTB, and ND2), the quinone reductase NQO2, and two key Parkinson's disease genes (PINK1 and UCHL1) figure within the enriched *T. gondii *interactome. While an ability of *T. gondii *to promote dopamine synthesis might be considered beneficial in Parkinson's disease, it has also been shown that dopamine promotes synuclein conformational changes, which may directly contribute to pathology [162]. As with other diseases, autoimmune networks, cancer pathways, and Alzheimer's disease were represented. Cancer and neurodegenerative diseases in general appear to be inversely correlated [163].

#### 4.5.2. Alzheimer's Disease

Any link between Alzheimer's disease and toxoplasmosis is limited to a seroprevalence study [13] and to scattered case reports [164, 165].

In Alzheimer's disease, the significantly enriched pathways included PPAR signalling, terpenoid biosynthesis (cholesterol synthesis) concerned with fatty acid, lipid, and cholesterol homoeostasis, and the arginine and proline metabolism pathway, primarily concerning nitric oxide, all of which play a key role in Alzheimer's disease physiology [166, 167]. 

Several pathogens (herpes simplex, *C. pneumoniae*, treponemas, and spirochetes) [24, 168, 169] increase beta-amyloid deposition. The gamma secretase network and APP are localised in immunocompetent dendritic cells, and, as the amyloid peptide possesses antimicrobial and antiviral effects [170, 171], beta-amyloid production may well be a general defensive response to pathogen invasion [20]. In normal conditions, it is not known whether beta-amyloid production is also a response to larger parasites, or whether beta-amyloid has antiparasitic activity.

In Tg2576 transgenic mice (the Swedish APP mutation), *T. gondii *infection in fact reduces cerebral beta-amyloid deposition and increases the levels of anti-inflammatory cytokines, effects attributed to the immunosuppressant effects of infection [172]. In relation to the cholesterol related genes in the Alzheimer's disease *T. gondii *dataset, the parasite cannot synthesis its own sterols and scavenges host cholesterol. Its growth in macrophages can be inhibited by statins [173]. While a living cholesterol lowering agent might be considered useful in the periphery, such effects may be deleterious if limited to cerebral areas, as the brain synthesises its own cholesterol. This is mostly present in myelin and is generally indispensable for function [167]. In the Alzheimer's disease Tg2576 transgenic model, *T. gondii *lysate antigen inhibits the production of nitrites in microglial cells, contributing to the protective effects of infection in this model [172]. As with obesity, certain interactome/susceptibility gene pathways involved in parasitic infection might well be considered as beneficial. 

#### 4.5.3. Multiple Sclerosis

Although by far the most enriched dataset in terms of interactome/susceptibility gene overlaps, there appear to have been no studies either in the clinic or in relation to myelination in laboratory studies linking multiple sclerosis and toxoplasmosis. A study in 3 pairs of identical twins reared apart was generally inconclusive, although *T. gondii* or other pathogen seropositivity were observed in some cases [174]. Further work will be of interest in relation to this close association. 

In multiple sclerosis, the major overlapping pathways primarily concerned cytokine and TGF-beta signaling, the related JAK-STAT pathway, and the ErbB and p53 signalling pathways that plays a key role in myelination [175, 176]. 

## 5. Summary

Within each disease dataset, the susceptibility genes that overlap with the *T. gondii *interactome, analysed by either method, appear highly relevant to the pathological processes and physiology of the disease. This convergence suggests a massive effect of infection on numerous processes. However, while some may be deleterious, (e.g., the promotion of dopaminergic activity in relation to psychosis), others may be beneficial (e.g., immunosuppression in autoimmune diseases). Even within any particular disease, the diverse effects of the parasite could be either favour or inhibit the development of particular endophenotypes. As suggested below, the overall direction taken and the resulting pathology are likely to depend upon a combination of factors including the strain of parasite, the timing and localisation of infection, our prior immune status, and the susceptibility genes. 

In many cases, the signalling networks influenced by susceptibility genes either *per se* or within the overlapping host/pathogen interactome involve many diseases other than the primary disease concerned. Diseases are often associated with other diseases, either positively or inversely [177]. For example degenerative disorders may be inversely associated with cancer [163, 178]. This may be related to particular signalling networks, for example growth factor signalling pathways are essential for myelination or involved in long-term potentiation, but excessive stimulation will promote cancerous growth. The ability of *T. gondii *(and other pathogens) to affect so many processes, which may be either deleterious or beneficial to various disease-related networks, suggests that pathogens may also be the pivot around which such relationships revolve.

### 5.1. Autoimmunity and Host/Pathogen Protein Homology

Several studies have recently shown that the entire human proteome contains short sequences (pentapeptides to heptapeptides or longer gapped consensi) that are identical to those within proteins expressed by numerous viruses, bacteria and other pathogens. For diverse pathogens, these human homologues appear to be concentrated within networks that are relevant to diseases in which the pathogen is implicated [35, 37, 38, 179, 180]. This problem is extensive and concerns all human proteins, along their entire length. For example, there are 18,000 pentapeptide overlaps between the poliovirus and the human proteome [181] while a single immunogenic pentapeptide (VGGVV) within the beta-amyloid peptide is identical to that within proteins from the herpes simplex virus and from 68 other viral species [19]. The extensive host/pathogen interactomes of numerous viruses, bacteria, and parasites no doubt result from this homology which enables pathogen proteins to mimic particular motifs within their human counterparts and to compete for their usual binding partners. Such homology must presumably relate to our evolutionary decent from monocellular organisms and to horizontal gene transfer, a process that applies to all living matter [182]. It is now also appreciated that DNA derived from both DNA and RNA viruses (and not only from retroviruses) has been extensively incorporated into the human genome, and it seems likely that this has also played a role in our evolution, and evidently in the generation of this protein homology [182–185]. Host parasite interactions have also contributed to this gene transfer, and genes from the Chagas disease vector, *Rhodnius prolixus*, have been found within the genomes of its tetrapod hosts [186]. Peptide homology is more extensive than genetic homology, due to the fact that a number of amino acids can be coded for by several triplet DNA codons (6 for arginine leucine and serine, 4 for alanine, glycine, proline, threonine, and valine, 3 for isoleucine, and 2 for asparagine, aspartate, glutamate, glutamine, cysteine, histidine, lysine, and phenylalanine) (see http://en.wikipedia.org/wiki/DNA_codon). These essentially correspond to single nucleotide polymorphisms that do not modify the translated amino acid. For short peptide sequences, numerous different DNA sequences can thus code for identical peptides.

This extensive homology, and more particularly slightly differing rather than identical peptides (which are more likely to be recognised as nonself) [36, 187], may well also contribute to autoimmunity problems that are evident in many diseases. For example, in Alzheimer's disease, multiple sclerosis, schizophrenia, and AIDS, antigenic regions of several autoantigens particular to each disease are homologous to proteins expressed by the pathogens implicated in the same disease (including *T. gondii *and schizophrenia) [18–21, 39].

Diseases currently classified as autoimmune include celiac disease, multiple sclerosis, myasthenia gravis, lupus, rheumatoid arthritis, and *inter alia* (see Medline Plus article at http://www.nlm.nih.gov/medlineplus/ency/article/000816.htm. However, the autoimmune problem appears to be much more extensive than currently appreciated. For example, using a protein array of 9,486 unique human protein antigens, even control blood samples averaged over 1000 autoantibodies, although with extreme intersample variation. As only ~30% of the human proteome was used in this experiment, we may each eventually accumulate over 3000 autoantibodies, irrespective of any particular disease. However, in both Parkinson's and Alzheimer's disease the target profile of the autoantibodies is distinct and can be reliably used as a diagnostic and predictive tool [188, 189]. Autoimmune signatures, with diagnostic predictive value have also been reported in multiple sclerosis [190], breast cancer [191], and nonsmall cell lung cancer [192]. Such data, (in diseases generally not regarded as autoimmune) and the recognition that so many diseases are characterised by immune activation and inflammation suggest that further research in this area would be fruitful in relation to the understanding of the pathologies and eventual treatment of many diseases.

The immune system is trained, in early life, not to recognize the body's own proteins as self [193]. These bioinformatics data suggest that the multiple autoantibodies seen in man (even in the absence of disease) may stem not from some inherent malfunction of the immune system itself, but from antibodies raised to the numerous pathogens that we randomly encounter during the course of our lifetime. Because of this extensive host/pathogen homology, such antibodies are also likely to target human proteins, and even if the pathogen is eliminated, continued encounter of these human homologues would sustain an autoimmune response. In this way, pathogens might be able to influence disease processes, even when no longer present, perhaps accounting for numerous studies that have failed to find pathogen DNA or protein within diseased tissue, a finding often cited as evidence against pathogen involvement, as recently applied to the controversial implication of the XMRV virus with chronic fatigue or prostate cancer [194–197]. The prospect that autoimmunity is pathogen related suggests that such agents may be able to punch far above their weight and influence biological processes even after their successful removal. This entails a revision of Koch's postulate as already discussed in a recent review on autoimmunity and the metagenome [177]. This autoimmune scenario might also explain why the antiparasitic agent artemisinin failed to influence psychotic symptoms (as add-on therapy) in schizophrenia [156], as destruction of the parasite needs not to affect the behaviour of antibodies raised to it.

Antibodies to pathogens are clearly cross-reactive with cerebral tissue, although the precise targets remain to be identified. For example 14/25 antibodies to 17 neurotropic pathogens, including *Borrelia burgdorferi*, *T. gondii*, and various DNA and RNA viruses were found to bind to western blots of human nervous tissue [198]. It is impossible to verify cross-reactivity solely from sequence analysis, but the ability of pathogen antibodies to react with human proteins could perhaps be tested in bulk using the protein arrays described above. It is now known that antibodies can enter cells, transported by the pathogens to which they bind, [199], and are also able to traverse the blood brain barrier [200]. Antibodies to receptors can also enter cells using the receptor endocytosis apparatus [201]. Antibodies can have devastating pathological consequences. For example, in transgenic mice engineered to express nerve growth factor antibodies only in lymphocytes, the blood brain barrier is soon disrupted, with cerebral antibody entry provoking extensive cortical degeneration, cholinergic neuronal loss, tau hyperphosphorylation, and beta-amyloid deposition (i.e., the cardinal pathology of Alzheimer's disease) [202]. This phenomenon is applicable to human diseases, including Sydenham's chorea, believed to be caused by *streptococcus* induced antibodies which cross-react with basal ganglia antigens [203]. The same streptococcal pathogens (and likely a similar mechanism) have been implicated in paediatric autoimmune neuropsychiatric disorders (PANDA's) whose diverse symptoms include tics, and dystonias, Tourette syndrome, and obsessive-compulsive disorder [204]. 

If autoantibodies do indeed play a key role in the pathogenesis of many diseases, then it is likely that their removal may be of benefit. However, given the large number of autoantibodies, some of which may well be beneficial and also required for pathogen defence, this may be no easy task. However, the research so far suggests that the number of autoantibodies specific to a particular disease may be more limited, allowing scope for analysis of their pathological or redemptive properties.

### 5.2. Population Genetics and a Proposed Gene/Environment Interaction Model (Figure 2)

The mechanisms described above provide a general example of multiple gene/environment interactions in relation to a single pathogen interactome, where several thousand genes (human and protozoan) are involved. Even for a simple population genetics model, with two genes, two risk factors, and a single cause, varying permutations can dramatically influence the eventual outcome. For example, the light and dark coloured genes of the peppered moth, or the light and dark colours of the clean or polluted trees on which they alight, can all be either risk promoting or protective depending on the varying permutations (the light gene “kills” the moth alighting on dark trees but is protective on the lighter trees, etc.) [205]. Neither gene, nor risk factor is relevant if there are no hungry birds or at night time. If one splits a complex disease into its component parts and gives the number of interacting processes involved in the *T. gondii *interactome, even this single pathogen could act either as a cause, a risk promoter, or as a protective agent, depending upon the pathways that it influences the most. For example, its effects on dopamine could promote psychosis or synuclein polymerisation, and its cholesterol scavenging may have beneficial effects in atherosclerosis, but deleterious effects on myelination, immunosuppressive effects might well protect against autoimmunity, but favour other infections, while the host's inflammatory reaction or associated fever might contribute to inappropriate collateral damage.

These complex interactions are nevertheless based on a relatively simple concept; that each interaction has an effect on the processes and pathways regulated by the human protein concerned. This suggests a model that may have general application to the many other pathogens and environmental agents implicated in these diseases.

If one imagines the *T. gondii *proteins as a number of spheres, each with particular affinity for certain human genes or proteins, and their human interactome partners as a further series of spheres perched on a genetic ledge whose characteristics and apertures are regulated by polymorphisms, mutations, deletions, translocations, or copy number variations, then the trajectory of each, dropped through this genetic sieve or knocked off the ledge and falling through the apertures, will be influenced both by the strain of pathogen with different host/pathogen affinities, the dropping point, the timing, and localisation of infection, when and where different human genes are expressed and by the polymorphic genes themselves (for both the host and the pathogen). 

Each of these human genes controls a particular element of one or many signalling networks, metabolic pathways, structural elements, developmental processes, and so forth, each represented by reception bins at different positions beneath the sieve. Depending upon varying permutations of these factors, the eventual number of spheres in each bin will vary, resulting in a diverse spectrum of pathway disturbance. Each pathway may be affected either positively or negatively, and the eventual assembly of this pathway mosaic leads to particular endophenotypes or subpathologies, which together combine to assemble into a particular disease. In this way, the same pathogen can produce diverse effects ranging from cause to prevention depending on a permutation of circumstance.

 The genes, risk factors, and the immune system thus work together to determine the final outcome, while neither *per se* are likely to provoke a particular disease. While gene/environment interactions are appreciated in both genetic and epidemiological studies, most, particularly in relation to GWAS, are performed without data partitioning in relation to other variables [206]. Many other pathogens (each no doubt with extensive host pathogen interactomes) and many other risk factors are implicated in these and other diseases, and many are able to influence several relevant aspects of pathology (see Section 1). A clearer understanding of these complex effects could perhaps result in a metamorphosis from multiple genes of small effect in large populations to more restricted numbers of greater effect in particular conditions. It is likely that many disease phenotypes have several “causes,” that subsets of overlapping genes are relevant to each, and that despite the mass of data collection and processing entailed, a dissection of these relationships could eventually lead to disease prevention and cure in multiple conditions.

By their very nature, polygenic diseases are complex, with several underlying pathologies and endophenotypes, hundreds of interacting genes, and dozens of environmental risk factors. The failure to replicate either genetic or epidemiological data is a situation peculiar to these diseases, not seen in many other fields. However, the effects of genes and risk factors are clearly conditional and, as illustrated above, may well depend upon each other. Replication inconsistency may well be part of the answer and not part of the problem. 

## 6. Conclusion

The host/pathogen interactome influences ~10% of the human genome products. This may seem a surprisingly high figure, but a similar interactome for the HIV-1 virus, maintained by NCBI http://www.ncbi.nlm.nih.gov/sites/entrez?Db=gene&Cmd=DetailsSearch&Term=hiv1interactions[properties], contains 1443 human genes (5.4% of the human genome). Bacteria and larger protozoan parasites, which, unlike viruses, also scavenge for host nutrients, as well as injecting their own metabolites into the host's environment, thus influence a larger spectrum of biochemical rather than signalling pathways. These data were also collected from experiments using various host (and species) tissues, and it is likely that brain or other tissue or time-specific interactomes would be more selective. 

The relevance of many genes to a particular condition is often tested by gene knockout in transgenic models and comforted by the resulting endophenotypes which mimic those of the particular disease [207]. However, risk promoting variants are, for the most part, single nucleotide polymorphisms rather than deletions and while expression may be altered (in either direction) in mRNA or protein expression studies, there is little to suggest a similar knockout in the human condition (see for example the microarray Geoprofiles database at NCBI http://www.ncbi.nlm.nih.gov/sites/geo/).

However, there are two pathogen-related effects that equate to conditional protein knockout which could be cell and regionally and temporally specific. The first relates to the host/pathogen interactome and the second to autoimmunity. If a host protein is engaged with that of a pathogen, it is effectively taken out of circulation during this period, and the pathways in which it is implicated can but be compromised. Secondly, because of extensive homology between pathogen and human proteins, antibody cross reactivity is likely to target the human counterparts of the pathogen antigen, effectively resulting in immunopharmacological knockout. In addition to these knockout effects, immune activation and the general reaction to infection are also likely to influence cellular function, as are the multitude of genes whose mRNA levels are influenced by this and other pathogens. In relation to prenatal effects, laboratory models have shown that maternally administered nonspecific viral DNA mimics and inflammatory agents or cytokines can also induce behavioural disturbances and psychopathology in the offspring [208, 209]. Fever during pregnancy also increases the risk of the offspring later developing autism and schizophrenia [210, 211], and it seems likely that prenatal infection in general is able to markedly affect brain development. The consequences would also depend upon which particular brain process and region is concerned at which period of embryogenesis.

Many other pathogens have been implicated in several of these conditions. In some of the diseases studied, almost one-third of the susceptibility genes were implicated in the *T. gondii *interactome (Table 3). Other pathogens will also have extensive interactomes, specific to each, but with a degree of overlap, and it would not be implausible if the near totality of susceptibility genes, in certain diseases, were involved in the summated life cycles of these diverse environmental triggers. It would thus seem that many susceptibility genes are related to the causes of disease, rather than (and as well as) to the disease itself. It is likely that stratification of GWAS and other genetic data in relation to infection status and history and many other environmental variables would be useful in determining the contribution of different genes to different risk factors and to their commonly affected pathways. 

Many psychiatric disorders are associated with a degree of social stigma and blame often apportioned to the genes, parentage and upbringing, and behaviour of the affected individuals. These and other chronic diseases also place a heavy long-term burden on family, friends, and caregivers [212]. This analysis suggests that *T. gondii *is a likely cause of certain aspects of psychiatric disorders, but perhaps a protective agent in others. Hopefully, an appreciation that such diseases may well be caused by pathogens and vectored by family pets will help to dispel such prejudice and more importantly create a new framework for the development of new methods of treatment and prevention. Given the massive problem of autoimmunity, however, it may be simplistic to suggest that removing the pathogen will halt the disease, although prevention of its initial access might be expected to affect disease incidence. Such approaches need not necessarily be clinical. For example if toxoplasmosis in cats and other pets was registered as a notifiable disease requiring obligatory treatment by veterinarians, perhaps the incidence of several diseases could be reduced. 

## Figures and Tables

**Figure 2:**
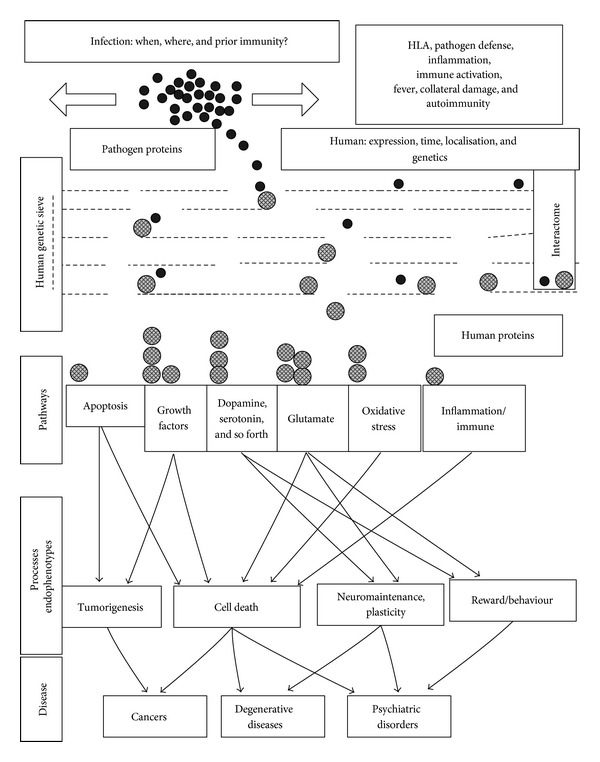
A model of the host pathogen interactome illustrating how multiple gene/environment interactions might direct the attentions of the pathogen towards distinct pathways, processes, and diseases. For any pathogen, immune and pathogen defence pathways as well as inflammatory processes will be activated to counter the infection. Although the pathogen can interact with hundreds of host genes and proteins, those chosen will depend upon the strain of pathogen, the timing and localisation of infection, and on whether prior immune barriers exist. In turn, which human elements are available for interaction will depend upon their expression (time and location) upon their functional quirks dictated by polymorphisms or mutations, and so forth. This selection process, involving a genetic sieve and individual interaction probabilities, enables similar interactome selectivity, allowing the pathogen to specifically affect different series of pathways in different circumstances (illustrated by the number of human proteins ending their route in a particular pathway bin). The differential modification of particular pathways will in turn affect particular processes and endophenotypes, whose final assembly constitutes the eventual mosaic of disease. This triage, involving both human and pathogen genes and proteins, as well as environmental factors, may explain how the same pathogen could cause, prevent, or otherwise influence a variety of diseases, depending upon genetic factors and a series of coincidences (see text for further details).

**Table 1:** Results of the KEGG pathway analysis of the *T. gondii *host/pathogen interactome (host genes): Immune and defence pathways, Diseases and other infections. The number of genes recovered in each pathway is in brackets and the enrichment *P* value from the CPDB analysis, provided where available, and significant values highlighted in bold.

	Number of genes	*P* value
**Immune and defence**		
Cytokine-cytokine receptor interaction	**(103)**	**2.02E** − **20**
Chemokine signalling pathway	**(64)**	**3.19E** − **09**
Toll-like receptor signalling pathway	**(52)**	**9.24E** − **17**
Phagosome	**(47)**	**1.02E** − **05**
Natural killer cell mediated cytotoxicity	**(46)**	**2.73E** − **07**
T cell receptor signalling pathway	**(45)**	**1.52E** − **10**
Hematopoietic cell lineage	**(42)**	**1.52E** − **12**
Leukocyte transendothelial migration	**(36)**	**0.000149**
NOD-like receptor signalling pathway	**(34)**	**9.21E** − **14**
Fc epsilon RI signalling pathway	**(29)**	**0.000764**
Fc gamma R-mediated phagocytosis	**(29)**	**0.00115**
Complement and coagulation cascades	**(28)**	**5.80E** − **07**
B cell receptor signalling pathway	**(28)**	**1.01E** − **05**
Lysosome	**(27)**	
Antigen processing and presentation	**(26)**	**5.34E** − **05**
Salivary secretion	(26)	
Adipocytokine signalling pathway	**(25)**	**0.000221**
RIG-I-like receptor signalling pathway	**(23)**	**0.000354**
Cytosolic DNA-sensing pathway	**(22)**	**0.000106**
Intestinal immune network for IgA production	**(22)**	**7.87E** − **07**

**Diseases**		
Pathways in cancer	**(94)**	**2.67E** − **08**
Transcriptional misregulation in cancer	**(52)**	**3.32E** − **06**
Prostate cancer	**(34)**	**6.94E** − **06**
Small cell lung cancer	**(31)**	**4.04E** − **06**
Colorectal cancer	**(23)**	**3.33E** − **05**
Pancreatic cancer	**(21)**	**0.0019**
Acute myeloid leukaemia	**(21)**	**8.14E** − **05**
Chronic myeloid leukaemia	**(20)**	**0.00747**
Glioma	**(19)**	**0.00964**
Renal cell carcinoma	(17)	
Endometrial cancer	**(16)**	**0.0048**
Non-small cell lung cancer	(15)	
Melanoma	(15)	
Bladder cancer	**(14)**	**0.00364**
**Neurological**		
Alzheimer's disease	**(52)**	**2.02E** − **05**
Huntington's disease	(34)	
Amyotrophic lateral sclerosis (ALS)	**(30)**	**7.94E** − **10**
Parkinson's disease	(27)	
Prion diseases	**(22)**	**2.26E** − **08**
**Autoimmune and atopicdiseases **		
Systemic lupus erythematosus	**(45)**	**2.48E** − **06**
Rheumatoid arthritis	**(44) **	**1.26E** − **12**
Type I diabetes mellitus	**(25)**	**1.11E** − **08**
Allograft rejection	**(23)**	**1.56E** − **09**
Autoimmune thyroid disease	**(22)**	**1.62E** − **05**
Graft-versus-host disease	**(21)**	**3.63E** − **06**
Asthma	**(14)**	**0.00422**
Primary immunodeficiency	**(13)**	**0.00168**
**Cardiac **		
Viral myocarditis	**(31)**	**1.24E** − **08**
Dilated cardiomyopathy	**(27)**	**0.00109**
Hypertrophic cardiomyopathy (HCM)	**(25)**	**0.00164**
Arrhythmogenic right ventricular cardiomyopathy (ARVC)	(18)	
**Other**		
Alcoholism	(40)	
Type II diabetes mellitus	**(19)**	**0.00194**
Maturity onset diabetes of the young	(2)	

**Other infections**		
HTLV-I infection	**(85)**	**1.62E** − **10**
Tuberculosis	**(80)**	**6.67E** − **19**
Influenza A	**(69)**	**5.11E** − **14**
Toxoplasmosis	**(66)**	**6.90E** − **20**
Herpes simplex infection	**(66)**	**6.88E** − **12**
Epstein-Barr virus infection	**(65)**	
Measles	**(56)**	**3.36E** − **13**
Amoebiasis	**(56)**	**4.59E** − **13**
Chagas disease (American trypanosomiasis)	**(55)**	**2.70E** − **16**
Pertussis	**(52)**	**1.38E** − **20**
Leishmaniasis	**(51)**	**2.49E** − **20**
Salmonella infection	**(47)**	**1.85E** − **14**
Hepatitis C	**(39)**	**0.000135**
Legionellosis	**(35)**	**9.83E** − **15**
Malaria	**(32)**	**2.90E** − **13**
Shigellosis	**(29)**	**4.94E** − **09**
Epithelial cell signalling in		
*Helicobacter pylori* infection	**(26)**	**1.83E** − **05**
Bacterial invasion of epithelial cells	**(26)**	**1.01E** − **05**
African trypanosomiasis	**(24)**	**1.56E** − **07**
*Staphylococcus aureus* infection	**(24)**	**7.63E** − **07**
Pathogenic *Escherichia coli* infection	**(21)**	**0.000147 **
*Vibrio cholerae* infection	(18)	

**Table 2:** Results of the KEGG pathway analysis of the *T. gondii *host/pathogen interactome: Signalling networks, Tissue and cellular process, metabolism and neuronal related pathways. The number of genes recovered in each pathway is in brackets and the enrichment *P* value from the CPDB analysis, provided where available, and significant values highlighted in bold.

	Number of genes	*P* value
**Signalling networks**		
MAPK signalling pathway	**(72)**	**8.43E** − **06**
Jak-STAT signalling pathway	**(59)**	**9.01E** − **12**
Calcium signalling pathway	**(44)**	**0.00797**
Insulin signalling pathway	(34)	
Wnt signalling pathway	(31)	
PPAR signalling pathway	**(29)**	**1.36E** − **05**
GnRH signalling pathway	(28)	
ErbB signalling pathway	**(26)**	**0.00146**
p53 signalling pathway	**(25)**	**1.83E** − **05**
VEGF signalling pathway	**(24)**	**0.0017**
TGF-beta signalling pathway	(19)	
Phosphatidylinositol signalling system	(19)	
mTOR signalling pathway	(15)	
Hedgehog signalling pathway	(7)	
Notch signalling pathway	(6)	

**Tissue process**		
Osteoclast differentiation	**(61)**	**7.00E** − **17**
Vascular smooth muscle contraction	(27)	
Bile secretion	(25)	
Melanogenesis	(25)	
Pancreatic secretion	(23)	
Mineral absorption	(22)	
Oocyte meiosis	(21)	
Carbohydrate digestion and absorption	**(20)**	**0.00207**
Protein digestion and absorption	(20)	
Endocrine and other factor-regulated calcium reabsorption	(19)	
Olfactory transduction	(17)	
Gastric acid secretion	(17)	
Aldosterone-regulated sodium reabsorption	**(16)**	**0.00283**
Progesterone-mediated oocyte maturation	(16)	
Cardiac muscle contraction	(13)	
Proximal tubule bicarbonate reclamation	(10)	
Vasopressin-regulated water reabsorption	(9)	
Taste transduction	(6)	
Vitamin digestion and absorption	(6)	
Collecting duct acid secretion	(4)	
Fat digestion and absorption	(4)	
Dorso-ventral axis formation	(4)	
Primary bile acid biosynthesis	(2)	
Renin-angiotensin system	(1)	

**Cellular process**		
Focal adhesion	**(56)**	**5.95E** − **06**
Cell adhesion molecules (CAMs)	**(50)**	**5.84E** − **10**
Regulation of actin cytoskeleton	**(49)**	**0.00475**
Apoptosis	**(45)**	**1.75E** − **15**
Endocytosis	(42)	
Protein processing in endoplasmic reticulum	(33)	
Extracellular matrix-receptor interaction	**(31)**	**2.29E** − **06**
ABC transporters	(23)	
Gap junction	(22)	
Cell cycle	(20)	
Ubiquitin mediated proteolysis	(20)	
Tight junction	(18)	
RNA transport	(16)	
Adherens junction	(16)	
Peroxisome	(15)	
Ribosome	(14)	
Regulation of autophagy	(11)	
Ribosome biogenesis in eukaryotes	(10)	
Spliceosome	(10)	
Proteasome	(10)	
RNA degradation	(8)	
RNA polymerase	(8)	
Base excision repair	(8)	
Nucleotide excision repair	(6)	
DNA replication	(5)	
Circadian rhythm: mammal	(4)	
Basal transcription factors	(3)	
Protein export	(3)	
mRNA surveillance pathway	(3)	
Mismatch repair	(2)	
SNARE interactions in vesicular transport	(2)	
Homologous recombination	(1)	

**Metabolism**		
Purine metabolism	**(53)**	**0.000397**
Pyrimidine metabolism	**(31)**	**0.00305**
Arginine and proline metabolism	**(24)**	**0.005**
Glycolysis/Gluconeogenesis	**(23)**	**0.0002**
Glutathione metabolism	**(21)**	**0.0008**
Arachidonic acid metabolism	(20)	
Glycerophospholipid metabolism	(20)	
Tryptophan metabolism	**(19)**	**0.002**
Oxidative phosphorylation	(19)	
Amino sugar and nucleotide sugar metabolism	(18)	
Inositol phosphate metabolism	(14)	
Fatty acid metabolism	(14)	
Galactose metabolism	(13)	
Valine, leucine and isoleucine degradation	(12)	
Glycine, serine and threonine metabolism	(12)	
Starch and sucrose metabolism	(12)	
Fructose and mannose metabolism	(12)	
Tyrosine metabolism	(12)	
Glycerolipid metabolism	(12)	
beta-Alanine metabolism	(11)	
Propanoate metabolism	(10)	
Glyoxylate and dicarboxylate metabolism	(10)	
Pyruvate metabolism	(9)	
Citrate cycle (TCA cycle)	(9)	
Drug metabolism, other enzymes	(8)	
Terpenoid backbone biosynthesis: (Cholesterol) Homo sapiens (human)	(8)	
Pentose phosphate pathway	(8)	
Nicotinate and nicotinamide metabolism	(8)	
Alanine, aspartate and glutamate metabolism	(8)	
Metabolism of xenobiotics by cytochrome P450	(8)	
Histidine metabolism	(7)	
Butanoate metabolism	(7)	
Cysteine and methionine metabolism	(7)	
Drug metabolism: cytochrome P450	(7)	
Steroid hormone biosynthesis	(7)	
Aminoacyl-tRNA biosynthesis	(7)	
**N** = **6**: One carbon pool by folate		
Biosynthesis of unsaturated fatty acids		
Linoleic acid metabolism		
Lysine degradation		
Ether lipid metabolism		
**N** = **5**: Sphingolipid metabolism		
N-Glycan biosynthesis		
Porphyrin and chlorophyll metabolism		
**N** = **4**: alpha-Linolenic acid metabolism		
Phenylalanine metabolism		
Retinol metabolism		
Synthesis and degradation of ketone bodies		
Fatty acid elongation		
Butirosin and neomycin biosynthesis		
Glycosaminoglycan degradation		
Steroid biosynthesis		
**N** = **3**: Glycosaminoglycan biosynthesis: chondroitin sulfate		
Pantothenate and CoA biosynthesis		
Glycosylphosphatidylinositol (GPI)-anchor biosynthesis		
Mucin type O-Glycan biosynthesis		
Pentose and glucuronate interconversions		
Selenocompound metabolism		
Ascorbate and aldarate metabolism		
D-Glutamine and D-glutamate metabolism		
**N** = **2**: Vitamin B6 metabolism		
Riboflavin metabolism		
Cyanoamino acid metabolism		
Glycosaminoglycan biosynthesis: heparan sulfate		
D-Arginine and D-ornithine metabolism		
Glycosphingolipid biosynthesis: ganglio series		
Folate biosynthesis		
Other types of O-glycan biosynthesis		
Caffeine metabolism		
Other glycan degradation		
Sulfur metabolism		
Glycosphingolipid biosynthesis: globo series		
**N** = **1**: Fatty acid biosynthesis		
Sulfur relay system Taurine and hypotaurine metabolism Lipoic acid metabolism Phenylalanine, tyrosine and tryptophan biosynthesis Lysine biosynthesis		
Ubiquinone and other terpenoid-quinone biosynthesis Glycosphingolipid biosynthesis: lacto and neolacto series: Glycosaminoglycan		
Biosynthesis: keratan sulfate		

**Neuronal**		
Neuroactive ligand-receptor interaction	(42)	
Dopaminergic synapse	**(39)**	**0.00284**
Neurotrophin signalling pathway	**(35)**	**0.00189**
Serotonergic synapse	(31)	
Glutamatergic synapse	(30)	
Cholinergic synapse	(28)	
Amphetamine addiction	**(27)**	**0.000172**
Retrograde endocannabinoid signalling	(25)	
Axon guidance	(24)	
Cocaine addiction	**(20)**	**0.000885**
Long-term potentiation	(19)	
Morphine addiction	(18)	
GABAergic synapse	(16)	
Long-term depression	(15)	
Synaptic vesicle cycle	(10)	
Nicotine addiction	(2)	
Phototransduction	(3)	

**Table 3:** A Statistical analysis of the overlap between human genes in the *T. gondii *Interactome, and the susceptibility genes in various diseases. The number of susceptibility genes analysed (*N* genes) is shown for each disease, together with the observed and expected values for each condition, the fold and mean enrichments, and the *P* value derived from the chi squared test.

Disease	*N* Genes	% involved in *T. gondii *interactome	Condition	Observed	Expected	Enrichment (fold)	Mean enrichment (A + B)/2	*P* value
Multiple Sclerosis	408	32.5	Susceptibility genes in interactome **(A)**	135	54.6	2.47	2.83	1.22*E* − 71
			Interactome genes in disease dataset **(B)**	135	42.4	3.18		
Alzheimer's	432	27.3	Susceptibility genes in interactome	118	57.8	2.04	2.33	2.26*E* − 41
			Interactome genes in disease dataset	118	44.9	2.63		
Schizophrenia	759	21.1	Susceptibility genes in interactome	160	101.6	1.57	1.80	3.06*E* − 27
			Interactome genes in disease dataset	160	78.9	2.03		
Bipolar disorder	443	21.2	Susceptibility genes in interactome	94	59.3	1.58	1.81	5.36*E* − 17
			Interactome genes in disease dataset	94	46.05	2.04		
Depression	221	23.5	Susceptibility genes in interactome	52	29.6	1.76	2.01	2.41*E* − 13
			Interactome genes in disease dataset	52	22.97	2.26		
Childhood obesity	73	31.5	Susceptibility genes in interactome	23	9.77	2.35	2.69	2.32*E* − 12
			Interactome genes in disease dataset	23	7.58	3.03		
Parkinson's disease	263	19.7	Susceptibility genes in interactome	52	35.21	1.47	1.69	3.82*E* − 08
			Interactome genes in disease dataset	52	27.34	1.90		
ADHD	237	17.7	Susceptibility genes in interactome	42	31.73	1.32	1.51	8.01*E* − 05
			Interactome genes in disease dataset	42	24.63	1.70		
Autism	1117	12.7	Susceptibility genes in interactome	142	149.55	0.95	1.08	0.013
			Interactome genes in disease dataset	142	116.13	1.22		
Anorexia	74	16.2	Susceptibility genes in interactome	12	9.91	1.21	1.38	0.09
			Interactome genes in disease dataset	12	7.69	1.55		
Chronic Fatigue	95	12.6	Susceptibility genes in interactome	12	12.72	0.94	1.08	0.48
			Interactome genes in disease dataset	12	9.87	1.21		

**Table 4:** The genes within each partition of  Figure 1 are annotated in the table below.

	Alzheimer's	Bipolar	Schizophrenia	Multiple sclerosis
Common to all	APOE GSK3B SYN3 **Cytokine** IL10 IL1B IL1RN IL6 TNF **Oxidative stress** GSTM1 ND4			

Alz, Bip, Sz	**Neuronal development/growth** DPYSL2 **Oxidative stress** MAOA NOS1 SOD2	**Neuronal development/growth** DPYSL2 **Oxidative stress** MAOA NOS1 SOD2	**Neuronal development/growth** DPYSL2 **Oxidative stress** MAOA NOS1 SOD2	

Alz, Bip and MS	**Chemokine** CCL2 **Oxidative stress** ND1	**Chemokine** CCL2 **Oxidative stress** ND1		CCL2 ND1

Bip, Sz and MS		**Immune** CTLA4 IFNG **Other **MMP9 PDE4B	**Immune** CTLA4 IFNG **Other **MMP9 PDE4B	**Immune** CTLA4 IFNG **Other **MMP9 PDE4B

Alz and Ms	**Immune** CCL3 CCR2 CD14 CD86 IL8 TAP2 TGFB1 **Oxidative stress** GSTM3 NOS2 **Other** APOC2 FAS GRN ICAM1 SERPINE1 TOMM40			APOC2 CCL3 CCR2 CD14 CD86 FAS GRN GSTM3 ICAM1 IL8 NOS2 SERPINE1 TAP2 TGFB1 TOMM40

Alz, Sz and MS	**Immune/inflammation** C4A PTGS2 Oxidative stress ATP6 CYTB **Other **CAV1 ESR1 MMP3 PPARG VDR		ATP6 C4A CAV1 CYTB ESR1 MMP3 PPARG PTGS2 VDR	ATP6 C4A CAV1 CYTB ESR1 MMP3 PPARG PTGS2 VDR

Bip and Sz		**Dopamine/glutamate/synaptic ** DRD2 DRD3 GRIN2A SYNGR1 TH **Signalling** FYN IMPA2 PIK3C3 PPP3CC **Growth** BMP6 CSF2RB EGR2 EGR3 **Circadian** PER3 **Other** ABCA13 ABCB1 ALOX12 BCL9 CIT DTNBP1 FABP7 GNL3 MLC1 MTHFD1 NAP5 NCAN PPARD TDO2 YWHAH	**Dopamine/glutamate/synaptic ** DRD2 DRD3 GRIN2A SYNGR1 TH **Signalling** FYN IMPA2 PIK3C3 PPP3CC **Growth** BMP6 CSF2RB EGR2 EGR3 **Circadian** PER3 **Other** ABCA13 ABCB1 ALOX12 BCL9 CIT DTNBP1 FABP7 GNL3 MLC1 MTHFD1 NAP5 NCAN PPARD TDO2 YWHAH	

Alz and Sz	**Cholesterol/lipoprotrein** ABCA1 LPL **Immune** C4B EBF3 IL18 IL1A **Other** KLF5 PCK1		ABCA1 C4B EBF3 IL18 IL1A KLF5 LPL PCK1	

Alz and Bip	HSPA5 **Growth** IGF1	HSPA5 **Growth** IGF1		

MS and SZ			**Immune **CCR5 CD4 CNTF HLA-A IGH@ IL12B IL2 IL4 LTA **Other **MYH9 PRKCA UCP2	**Immune **CCR5 CD4 CNTF HLA-A IGH@ IL12B IL2 IL4 LTA **Other **MYH9 PRKCA UCP2

	Specific to Alzheimer's: **APP processing:** APP APBB1 APBB2 APH1B ADAM10 GAPDH PSENEN **Cholesterol/lipoprotein/PPAR **APOD CH25H FDPS HMGCR HMGCS2 LDLR LRP1 MMP1 NPC2 OLR1 PPARA SOAT1 **Complement/immune/cytokine** A2M CD2AP CD33 CD36 CR1 CRP CSF1 F13A1 IL33 LCK PLAU PLTP SERPINA1 TAPBPL TLR2 TLR4 **Oxidative stress **COX3 HMOX1 NFE2L2 **Apoptosis **CTSD NLRP1 **Ubiquitin** UBD UBE2I UCHL1 **Other:** ACAN AHSG ALB ARSB CAND1 CDC2 CECR2 FAM63A GBP2 HSPG2 LMNA MTHFD1L OTC PARP1 PCMTD1 PDE9A PVRL2 RBL1 SASH1 SCN2A SEL1L SGPL1 SSB TTLL7 ZBP1	Specific to Bipolar disorder: **Monoamine/GABA** DDC DRD1 GABRB3 GCHI **Signalling** AKT1 CREB1 DUSP6 PLCG1 TEC **Adhesion** CD276 CDH20 SDC2 **Lysosome** CTSH LAMP3 **Ion channel/transport**: SCN8A SLC12A6 SLC26A7 TRPM2 **Other:** ATF3 BDKRB2 COLEC12 DPP10 DPY19L3 FAM115A FKBP5 FOXN3 GPX3 HK2 HNRNPC HSP90B1 LRRC36 MCM3APAS N6AMT1 NR1D1 PLSCR4 SNX27 STAB1 SVEP1 TLE4 TSHZ2	Specific to schizophrenia: **Monoamine** ADRA1A ALDH1A2 DRD4 DRD5 HTR3E PHOX2A SLC6A3 **Glutamate** DLG2 DLG4 HOMER1 NAALAD2 SLC1A3 SRR **Other transmitters**: ADORA1 CNP NPY PDYN VIPR2 **Neuregulin/growth factor** CSPG5 EGR4 ERBB2 GFRA3 NRG2 PDGFB **Complement/immune/cytokine **C3 CFB HLA-DQA2 IFT88 IL10RA IL18R1 IL3 IL3RA LIF SLAMF1 TNFRSF1B **Glutathione/oxidative stress** GCLC GCLM GSS NQO2 SEPSECS **Adhesion** CHL1 CNTN1 FLNB GLG MAG PDCD1LG2 **Signalling** ARHGAP18 ARHGEF10 ATM MAPK14 NFKB1 PLA2G4A PPP3CB PTPRZ1 RELA SFRP1 TCF7L2 TNIK **Transporters Na** ^+^ **/K** ^+^ **/Cl** ^−^ SLC12A2 **Zinc/cadmium** SLC39A8 **Iron** SLC40A1 **Neuronal migration/development ** NDE1 PAFAH1B1 PLXNA2 **Other:** ADA AGAP1 ANXA1 ATXN3 CALR CHN2 DNMT3B ERC2 FOLH1 GPC1 PAX6 PNPO RANBP1 RHD SIGMAR1 SMARCA2 TGM2 TSPO TXNDC5 UFD1L	Specific to multiple sclerosis: **Complement/immune/cytokine **C5 C7 CCL1 CCL11 CCL14 CCL5 CCL7 CD226 CD24 CD28 CD40 CIITA CXCL10 CXCL12 CXCR4 CXCR5 ERAP1 FCGR3B ICOS I IFI30 IFIT1 IFNGR2 IL12A IL2RA IL4R IL7 IL7R IRF1 IRF8 MIF MX1 NOD2 PDCD1 PRF1 PTGER4 PVR SLC11A1 SPP1 TNFRSF1A TNFSF10 TRB@ TRD@ TYK2 CYP24A1 (Vitamin D) **Signalling** CDC37 CHUK JAG1 MAPK1 MYC NFKBIA PLCL1 PTPN2 RPS6KB1 SOCS1 STAT1 **Oxidative stress** DDAH1 NDUFS5 NDUFS7 **Apoptosis** CASP8 CASP9 **Metalloproteases** MMP2 MMP12 **Other:** ACTN1 ANKRD55 FAM164A GPC5 ITGAM LAG3 METTL1 MPHOSPH9 PSMB8 PSMB9 PSORS1C1 PTAFR RGS1 SLC25A36 TAC1 WDYHV1 ZIC1 ZNF532

**Table 5:** Significantly enriched KEGG pathways within the overlapping interactome/susceptibility gene datasets (Immune related, other diseases and infections). *P* values from the CPDB analysis are provided after each pathway description.

	Immune and defence	Diseases	Other infections
ADHD	None	None	None

Autism	Intestinal immune network for IgA production 0.00138 Hematopoietic cell lineage 0.00196 T cell receptor signalling pathway 0.00439 Fc epsilon RI signalling pathway 0.00838	Dilated cardiomyopathy 0.000293 Arrhythmogenic right ventricular cardiomyopathy (ARVC) 0.000899 Hypertrophic cardiomyopathy (HCM) 0.00151 Viral myocarditis 0.00548	Leishmaniasis 0.00605

Anorexia	None	None	None

Childhood obesity	Intestinal immune network for IgA production 0.00417 NOD-like receptor signalling pathway 0.00604	Graft-versus-host disease 0.0000725 Type I diabetes mellitus 0.0000837 Transcriptional misregulation in cancer 0.000397 Rheumatoid arthritis 0.000774 Allograft rejection 0.0025 Type II diabetes mellitus 0.00417 Alzheimer's disease 0.00426	Chagas disease (American trypanosomiasis) 0.00114 African trypanosomiasis 0.00223 Malaria 0.0047 Legionellosis 0.00544 Herpes simplex infection 0.0056 Pertussis 0.00968

Depression	Cytokine-cytokine receptor interaction 0.0000494 NOD-like receptor signalling pathway 0.000203 Hematopoietic cell lineage 0.000998	Rheumatoid arthritis 0.000006 Graft-versus-host disease 0.0000516 Alzheimer's disease 0.000173 Allograft rejection 0.000845	Leishmaniasis 0.0000000000696 Malaria 0.000000000132 African trypanosomiasis 0.000000000337
	Intestinal immune network for IgA production 0.00181 T cell receptor signalling pathway 0.00199 Antigen processing and presentation 0.00665 Fc epsilon RI signalling pathway 0.00741	Type I diabetes mellitus 0.00131 Amyotrophic lateral sclerosis (ALS) 0.00215 Pathways in cancer 0.006 Hypertrophic cardiomyopathy (HCM) 0.00848 Small cell lung cancer 0.00965	Chagas disease (American trypanosomiasis) 0.00000000199 Tuberculosis 0.0000000149 Amoebiasis 0.0000000505 Legionellosis 0.00000786
			Influenza A 0.0000249 Herpes simplex infection 0.0000333 Pertussis 0.0000338 Toxoplasmosis 0.0000548 Salmonella infection 0.000916 HTLV-I infection 0.00202 Measles 0.00463

Bipolar disorder	T cell receptor signalling pathway 0.0000000071 NOD-like receptor signalling pathway 0.0000794 Cytokine-cytokine receptor interaction 0.000951 Antigen processing and presentation 0.00275 Fc epsilon RI signalling pathway 0.00317 Natural killer cell mediated cytotoxicity 0.00379 Toll-like receptor signalling pathway 0.00784	Prostate cancer 0.0000601 Pathways in cancer 0.000895 Osteoclast differentiation 0.00000583 Amyotrophic lateral sclerosis (ALS) 0.0000424 Rheumatoid arthritis 0.0000681 Prion diseases 0.000142 Graft-versus-host disease 0.000266 Alzheimer's disease 0.000268 Allograft rejection 0.00283 Type I diabetes mellitus 0.00435	Malaria 0.000000108 Tuberculosis 0.000000925 Chagas disease (American trypanosomiasis) 0.00000122 African trypanosomiasis 0.00000646 Measles 0.0000734 HTLV-I infection 0.000178 Influenza A 0.000371 Amoebiasis 0.00132 Leishmaniasis 0.00226 Pertussis 0.0025 Herpes simplex infection 0.00273 Toxoplasmosis 0.00368 Legionellosis 0.00867

Schizophrenia	Cytokine-cytokine receptor interaction 0.000000000286 T cell receptor signalling pathway 0.000000000429	Type I diabetes mellitus 0.00000000983 Allograft rejection 0.0000000512 Graft-versus-host	Leishmaniasis 0.0000000000322 Tuberculosis 0.000000000102 Pertussis 0.000000000705
	NOD-like receptor signalling pathway 0.0000216 Hematopoietic cell lineage 0.0000452 Fc epsilon RI signalling pathway 0.000161 Adipocytokine signalling pathway 0.00053 Intestinal immune network for IgA production 0.000682	disease 0.000000121 Rheumatoid arthritis 0.00000747 Amyotrophic lateral sclerosis (ALS) 0.000009 Asthma 0.00007 Autoimmune thyroid disease 0.0001 Alzheimer's disease 0.0007	African trypanosomiasis 0.000000032 Chagas disease (American trypanosomiasis) 0.000000038 HTLV-I infection 0.000000319 Salmonella infection 0.000000504
	Toll-like receptor signalling pathway 0.000773 Antigen processing and presentation 0.000887 Cytosolic DNA-sensing pathway 0.00218 Natural killer cell mediated cytotoxicity 0.00387 RIG-I-like receptor signalling pathway 0.00395 B cell receptor signalling pathway 0.00472	Systemic lupus erythematosus 0.0009 Transcriptional misregulation in cancer 0.001 Prion diseases 0.001 Prostate cancer 0.002 Pathways in cancer 0.003 Acute myeloid leukaemia 0.0099	Measles 0.000000634 Legionellosis 0.00000129 Influenza A 0.0000017 Toxoplasmosis 0.00000446 Herpes simplex infection 0.0000156 Amoebiasis 0.0000259 Malaria 0.000099 *Staphylococcus aureus* infection 0.000152 Viral myocarditis 0.00372

Multiple sclerosis	Cytokine-cytokine receptor interaction 1.02E*‒*28 Toll-like receptor signalling pathway 0.000000000000000132	Allograft rejection 0.000000000000000147 Type I diabetes mellitus 0.0000000000000549 Rheumatoid arthritis	Chagas disease (American trypanosomiasis) 5.89*E* − 22 Influenza A 2.81*E* − 19 Toxoplasmosis 4.01*E* − 18
		Chemokine signalling pathway 0.000000000000747 Intestinal immune network for IgA production 0.000000000007 T cell receptor signalling pathway 0.0000000000237	0.000000000000153 Graft-versus-host disease 0.0000000000336 Autoimmune thyroid disease 0.000000000425 Pathways in cancer 0.0000000102
	NOD-like receptor signalling pathway 0.0000000000639 Hematopoietic cell lineage 0.000000000469	Systemic lupus erythematosus 0.000000625 Prion diseases 0.00000316 Alzheimer's disease 0.00000422	Pertussis 0.000000000000006 Herpes simplex infection 0.0000000000000315
	Natural killer cell mediated cytotoxicity 0.00000058 RIG-I-like receptor signalling pathway 0.00000188	Transcriptional misregulation in cancer 0.00000875 Asthma 0.0000253 Small cell lung cancer 0.000078	African trypanosomiasis 0.00000000000585 Malaria 0.0000000000143
	Primary immunodeficiency 0.00000316 Antigen processing and presentation 0.0000325 Leukocyte transendothelial migration 0.0000719	Colorectal cancer 0.0000921 Bladder cancer 0.000135 Amyotrophic lateral sclerosis (ALS) 0.000341 Prostate cancer 0.00067	Amoebiasis 0.0000000000237 Legionellosis 0.0000000000346 Viral myocarditis 0.0000000089 Salmonella infection 0.0000000677
	Cytosolic DNA-sensing pathway 0.0000921 Complement and coagulation cascades 0.00927 Adipocytokine signalling pathway 0.00927	Pancreatic cancer 0.00147 Chronic myeloid leukaemia 0.00177 Endometrial cancer 0.00339 Acute myeloid leukaemia 0.00473 Thyroid cancer 0.00487	HTLV-I infection 0.000000512 Hepatitis C 0.000000538 *Staphylococcus aureus* infection 0.00000371 Shigellosis 0.000786 Epithelial cell signalling in *Helicobacter pylori* infection 0.00882

Alzheimer's	Hematopoietic cell lineage 0.000000276 Complement and coagulation cascades 0.00000049 Toll-like receptor signalling pathway 0.000000982 NOD-like receptor signalling pathway 0.000002 Cytokine-cytokine receptor interaction 0.000003	Rheumatoid arthritis 0.0000000000000007 Alzheimer's disease 0.000000013 Graft-versus-host disease 0.0000035 Type I diabetes mellitus 0.000076 Prion diseases 0.000442 Transcriptional misregulation in cancer 0.00049	Malaria 6.05E *‒* 17 Chagas disease (American trypanosomiasis) 0.00000000004 Pertussis 0.000000000204 Leishmaniasis 0.00000000283 Tuberculosis 0.00000000325 Legionellosis 0.00000000407
	Cytosolic DNA-sensing pathway 0.000436 Phagosome 0.00098 Intestinal immune network for IgA production 0.00148 Adipocytokine signalling pathway 0.00558	Allograft rejection 0.000549 Hypertrophic cardiomyopathy (HCM) 0.00165 Pathways in cancer 0.00205 Systemic lupus erythematosus 0.00281	Influenza A 0.0000000246 African trypanosomiasis 0.0000000547 Amoebiasis 0.000000126 Salmonella infection 0.000000226

Parkinson's	NOD-like receptor signalling pathway 0.00000539 Toll-like receptor signalling pathway 0.00000543 Hematopoietic cell lineage 0.0000417 Cytokine-cytokine receptor interaction 0.000153 T cell receptor signalling pathway 0.00123 Intestinal immune network for IgA production 0.00125 Cytosolic DNA-sensing pathway 0.00261 Complement and coagulation cascades 0.00354 Antigen processing and presentation 0.00465 Chemokine signalling pathway 0.00977	Rheumatoid arthritis 0.000000131 Graft-versus-host disease 0.000000932 Type I diabetes mellitus 0.00000119 Asthma 0.00031 Systemic lupus erythematosus 0.000327 Prion diseases 0.000491 Allograft rejection 0.00058 Alzheimer's disease 0.000792 Parkinson's disease 0.00261 Pathways in cancer 0.00315 Hypertrophic cardiomyopathy (HCM) 0.00594 Small cell lung cancer 0.00677 Prostate cancer 0.00721	Measles 0.000463 Toxoplasmosis 0.000463 Herpes simplex infection 0.000578 *Staphylococcus aureus* infection 0.00246 Pertussis 0.000000000000612 Tuberculosis 0.00000000000987 Leishmaniasis 0.0000000254 Influenza A 0.0000000563 Malaria 0.000000086 Salmonella infection 0.0000000885 Legionellosis 0.000000137 Chagas disease (American trypanosomiasis) 0.00000033 Amoebiasis 0.000000376 African trypanosomiasis 0.000000412 *Staphylococcus aureus* infection 0.0000997 Measles 0.000305 Toxoplasmosis 0.000305 Hepatitis C 0.00291 HTLV-I infection 0.00604 Herpes simplex infection 0.00858

**Table 6:** Significantly enriched KEGG pathways within the overlapping interactome/susceptibility gene datasets (Signalling networks, metabolic pathways and neuronal pathways). *P* values are provided after each pathway description.

	Signalling networks	Process	Metabolism	Neuronal
ADHD	Calcium signalling pathway 0.00442		Histidine metabolism 0.000162 Tryptophan metabolism 0.000473 Phenylalanine metabolism 0.00213 Biosynthesis of unsaturated fatty acids 0.0029 Tyrosine metabolism 0.00932	Dopaminergic synapse 5.29*E* − 07 Cocaine addiction 7.07*E* − 07Neuroactive ligand-receptor interaction 0.000559

Autism	VEGF signalling pathway	ECM-receptor interaction 0.000918 Cell adhesion molecules (CAMs) 0.00109 Focal adhesion 0.00239	None	Cocaine addiction 0.000086 Amphetamine addiction 0.00061 Dopaminergic synapse 0.00203 Serotonergic synapse 0.0079

Anorexia	None	None	None	Dopaminergic synapse 0.0000132 Cocaine addiction 0.000019 Neuroactive ligand-receptor interaction 0.000239 Amphetamine addiction 0.00296 Morphine addiction 0.00501 Serotonergic synapse 0.00924

Childhood obesity	PPAR signalling pathway 0.000011 p53 signalling pathway 0.00822	None	Glycerolipid metabolism 0.00525	None

Depression	Calcium signalling pathway 0.000239 Circadian rhythm—mammal 0.00546 VEGF signalling pathway 0.00618 Jak-STAT signalling pathway 0.00772 TGF-beta signalling pathway 0.00877	Osteoclast differentiation 0.000453 Apoptosis 0.000876 Gap junction 0.00104 Melanogenesis 0.00166	Tryptophan metabolism 0.0000468 Arginine and proline metabolism 0.00296 Phenylalanine metabolism 0.00366 Biosynthesis of unsaturated fatty acids 0.00498 alpha-Linolenic acid metabolism 0.00498 Histidine metabolism 0.00876	Dopaminergic synapse 0.000000000736 Cocaine addiction 0.0000000875 Amphetamine addiction 0.00000108 Serotonergic synapse 0.0000323 Morphine addiction 0.00104 Retrograde endocannabinoid signalling 0.00155 Neuroactive ligand-receptor interaction 0.00244 Glutamatergic synapse 0.00351 Long-term depression 0.00529

Bipolar disorder	Calcium signalling pathway 0.000384 Jak-STAT signalling pathway 0.00121	Apoptosis 0.0000464	Tyrosine metabolism 0.00306 Tryptophan metabolism 0.00354 Phenylalanine metabolism 0.00828	Dopaminergic synapse 0.00000000379 Cocaine addiction 0.0000000438 Amphetamine addiction 0.000000813 Neurotrophin signalling pathway 0.00292 Neuroactive ligand-receptor interaction 0.00496

Schizophrenia	Jak-STAT signalling pathway 0.00000299 MAPK signalling pathway 0.000558 VEGF signalling pathway 0.00077 PPAR signalling pathway 0.00395 Calcium signalling pathway 0.00444 Wnt signalling pathway 0.00661	Apoptosis 0.000000043 Osteoclast differentiation 0.00000285 Cell adhesion molecules (CAMs) 0.000787	Glutathione metabolism 0.00583	Cocaine addiction 0.0000000151 Dopaminergic synapse 0.0000000571 Amphetamine addiction 0.00000666 Glutamatergic synapse 0.000494 Serotonergic synapse 0.00944

Multiple sclerosis	Jak-STAT signalling pathway 0.000000000000259 TGF-beta signalling pathway 0.000492 MAPK signalling pathway 0.00126 ErbB signalling pathway 0.00382 p53 signalling pathway 0.00882	Osteoclast differentiation 0.000000000314 Cell adhesion molecules (CAMs) 0.0000000558 Apoptosis 0.0000000604		

Alzheimer's	PPAR signalling pathway 0.0000000416	Osteoclast differentiation 0.000351	Terpenoid backbone biosynthesis 0.00125 Arginine and proline metabolism 0.0028	

Parkinson/s	MAPK signalling pathway 0.00623	Apoptosis 0.000537 Osteoclast differentiation 0.00246	Arginine and proline metabolism 0.00205 Histidine metabolism 0.00684	Dopaminergic synapse 0.0000000876 Cocaine addiction 0.000045 Amphetamine addiction 0.0034
